# Dietary Bile Acid Supplementation Could Regulate the Glucose, Lipid Metabolism, and Microbiota of Common Carp (*Cyprinus carpio* L.) Fed with a High-Lipid Diet

**DOI:** 10.1155/2023/9953927

**Published:** 2023-05-24

**Authors:** Liping Yang, Mingyu Liu, Mengjuan Zhao, Shaoyang Zhi, Wenlei Zhang, Leya Qu, Jinrui Xiong, Xiao Yan, Chaobin Qin, Guoxing Nie, Shengpeng Wang

**Affiliations:** ^1^College of Fisheries, Henan Normal University, No. 46 Jianshe Road, Xinxiang 453007, China; ^2^Dezhou Key Laboratory for Applied Bile Acid Research, Shandong Longchang Animal Health Product Co., Ltd., Dezhou, China

## Abstract

This study sought to examine the role of bile acids in the regulation of glucose and lipid metabolism, intestinal flora, and growth in high-fat diet-fed common carp (*Cyprinus carpio* L.). Fish (6.34 ± 0.07 g) were fed for 56 days with three different diets, the control diet (CO, 5.4% lipid), high-fat diet (HF, 11% lipid), and high-fat diet with 60 mg/kg bile acids (BAs, 11% lipid). The results showed that high-fat diets resulted in poor growth performance and increased triglyceride (TG) in serum and the liver. The addition of bile acids significantly alleviated the adverse effects of a high-fat diet. The mRNA expression results indicated that bile acids may improve lipid metabolism through the enhancement of the peroxisome proliferator-activated receptor (PPAR*a*). The expression of gluconeogenesis-related phosphoenolpyruvate carboxykinase (PEPCK) mRNA was inhibited, while fibroblast growth factor 19 (FGF19) was significantly higher. Bile acids reshaped the intestinal microflora community, with the level of *Bacteroidetes* increasing. The correlation analysis indicated that *Patescibacteria*, *Dependentiae*, *Myxococcota,* and *Planctomycetota* in the gut are associated with genes involved in glucose and lipid metabolism. These results indicated that bile acids could ameliorate the negative effects of high-fat diets on common carp.

## 1. Introduction

Lipids are one of the main macronutrients needed for fish development and growth, and they are the energy source in fish feed. Fat in the diet is able to supply essential fatty acids, phospholipids, and cholesterol. Moreover, fat in the diet also plays a role in increasing the absorption of lipophilic nutrients, which could contribute to the flavor and quality of fish [1]. Interestingly, nonprotein energy nutrients such as lipids are widely used to save protein due to their effects on protein sparring [2]. Therefore, appropriate lipids in the diet could be beneficial for fish growth due to the effective utilization of protein [3]. However, it has been reported that the long-term excessive intake of a high-fat diet might induce obesity and lead to glycolipid metabolism disorder and even insulin resistance in female C57BL/6 mice, common carp (*Cyprinus carpio* L.) and Nile tilapia (*Oreochromis niloticus*) [4–6]. In much more severe cases, it could lead to nonalcoholic fatty liver disease, which could induce metabolic disorders and trigger healthy diseases [7].

Bile acids (BAs) are amphiphilic sterol compounds that contain hydroxyl or carboxyl groups and alkyl groups, which are mainly synthesized from cholesterol by the liver [8]. After synthesis, BAs are stored in the gallbladder, and they are emptied into the intestine when needed. They could also be reabsorbed efficiently in the intestine and transported back to the liver for recirculation. Bile acids play important roles in emulsification, fat metabolism, facilitation of the digestion, absorption of lipids and lipid-soluble nutrients [9–11], and in cholesterol homoeostasis [12, 13]. In addition, bile acids are also known as signaling molecules that regulate their own energy and glucose homeostasis [14]. Therefore, bile acids are widely used to treat metabolic diseases [15]. Moreover, BAs were developed to alleviate fat accumulation and improve growth performance in grass carp (*Ctenopharyngodon idella*), tiger puffer (*Takifugu rubripes*), and *Micropterus salmoides* [16–18]. It has been reported that the effective concentration of BAs is 60 mg/kg in common carp [19].

One of the natural ligands of bile acids is the farnesol X receptor (FXR), a nuclear transcription factor that is widely distributed in the gallbladder, liver, ileum, kidney, and other organs [20]. In recent years, studies have shown that BAs act as signaling factors to affect glucose metabolism through FXR [21], and FXR is a key regulator of bile acid homeostasis and enterohepatic circulation. Bile acids activate FXR and induce the formation of small heterodimer partners (SHPs) to secrete bile acids into the small intestine. At the same time, the transcription of fibroblast growth factor 19 (FGF19) is activated, and then the activities of cholesterol 7 alpha-hydroxylase (CYP7A1), and sterol 12 alpha-hydroxylase (CYP8B1), which are the rate-limiting enzymes in bile-acid synthesis, are inhibited. The activation of FGF19 reduces the expression of cAMP response element binding protein (CREB), glucose-6-phosphatase (G6Pase), and phosphoenolpyruvate carboxykinase (PEPCK) (gluconeogenesis); therefore, blood glucose is downregulated [22, 23]. This mechanism has been verified in mice [24]. Although the inhibition of hepatic glucose synthesis by activation of FXR has been demonstrated in mammals, it has been less well studied in fish.

The bacterial community in the gut critically responds to host health and is a key environmental factor in the pathogenesis of liver diseases [25]. Bile acids cannot only act as signaling molecules to regulate energy metabolism in the body but also have strong antibacterial effects [26, 27]. Bile acids regulate the intestinal microbiota and inhibit the proliferation of harmful bacteria such as *Escherichia coli* and *Enterococcus faecalis* in the intestine [28].

The common carp is a domesticated freshwater fish that is widely farmed in China. According to the China Fishery Statistical Yearbook 2021, the production of common carp reached 2.896 million tons in 2020. Although the yield of carp is very high, it is also plagued by metabolic diseases, leading to a reduction in growth indicators. In this study, common carp were set as the control group with a 5.4% fat normal diet, 11% fat high-fat diet, and 11% fat high-fat diet supplemented with 60 mg/kg bile acids (Shandong Longchang Bile Acids). A 56-day culture experiment was carried out to investigate the effects of bile acids on lipids, glucose metabolism, intestinal flora, and the growth of common carp. The results from the culture experiment will indicate the function of bile acids on the glycolipid metabolism and the community of gut microbiota in common carp after being fed a high-fat diet and supplemented with bile acids.

## 2. Methods and Materials

### 2.1. Experimental Animal and Diets

The experiment was carried out in the recirculation aquaculture system at the Henan Normal University. The juvenile common carp was provided by Zhengzhou Xingda Breeding Company (Henan, China). Fish was acclimated in 300 L tanks with a commercial diet. After the 2 weeks of acclimation, the similarly sized fish (6.34 ± 0.07 g) was weighted and selected in the breeding experiment. And they were randomly assigned to 9 tanks, each tank with 30 fish. The handling and operation of fish complied with the guidelines of Henan Normal University institutional care and use of animals.

Three isonitrogenous but nonisolipidic tested diets were designed and shown in Table 1. Basic diets with a suitable lipid (5.4%) to meet the nutritional needs of common carp were used as the control diet (CO). High lipid diets (HF, 11%) were prepared by adding more corn oil than basic diets. Bile acids (>950 g kg^−1^ purity) extracted from porcine bile containing 69% hyodeoxycholic acid, 9% hyocholic acid, 19% chenodeoxycholic acid, and trace ursodeoxycholic acid and binding bile acids were provided from Longchang Animal Health Products Co., Ltd. (Jinan, Shandong Province, China). BAs diets were formulated by adding 60 mg/kg of bile acids into the HF diets (BA group) according to Yao et al. [19]. The mixture, extrusion, packaging, and storage of the diets followed the previous report of our laboratory [29].

### 2.2. Feeding Trial

The feeding trial lasted for 56 days, and all fish were fed three times daily to apparent satiation during the experimental period (9 : 00, 14 : 00, and 18 : 00). When the about 4 granules kept sinking into the bottom for more than 10 mins, or when each feeding time exceeds 1 hour, the fish is considered to have reached satiation. The dissolved oxygen concentrations were between 5.1~6.1 mg/L, and the pH was 7.2~7.7. The water temperature was 26 ± 1°C. The photoperiod was 12-h light and 12-h darkness.

### 2.3. Sample Collection

At the end of the 56-day feeding trial, all fish were fasted for 24 h before the fish were anesthetized (MS-222, 55 mg/L). All fish in each tank were counted and weighed. The weights of the fish, liver, viscera, and body length of the fish were all measured. Then, the visceral somatic index, hepatopancreas somatic index, and condition factor were computed. Blood was taken from the caudal vein of common carp using a syringe with a 25-gauge needle. The serum samples were obtained by centrifuging at 4,000 rpm for 10 min. The liver, muscle, and intestine tissues were sampled on ice and were stored at −80°C for further analysis. The liver was cut into 2 cm pieces for tissue fixation. The intestine was firstly removed from the common carp under sterile environments, and then the hindgut contents were carefully squeezed out and collected for subsequent DNA extraction.

### 2.4. Analytical Method

#### 2.4.1. Proximate Analysis

Chemical analyses of the experimental diets, whole fish, and muscle were determined according to a Weende analysis method. The measurement of moisture was based on the national standards in China (GB 5009.3–2016). The samples were dried to stable weight at 105°C. The analyses of the ash content were according to the national standards in China (GB 5009.4–2016), with the samples burning in a muffle furnace till they were ashed completely at 550°C. The contents of crude protein and lipid were determined, respectively, by using a Kjeltec method and the Soxhlet extractor method.

#### 2.4.2. Biochemical Parameters in Serum and Liver

Serum glucose (GLU), triglyceride (TG), total cholesterol (TC), total bile acids (TBA), low–density lipoprotein cholesterol (LDL-C), and high-density lipoprotein cholesterol (HDL-C) were quantified by using the specific commercial kits supplied by Jiancheng Bioengineering Institute in Nanjing, China.

The level of TC, TG, HDL-C, and LDL-C and the concentration of TBA were measured using the supernatant of common carp liver which was homogenized in an ice bath in absolute alcohol. The supernatants were achieved by centrifuging at 3000 rpm for 10 min at 4°C.

#### 2.4.3. RNA Extraction and Real-Time Quantitative PCR (RT-qPCR)

The total RNA of the liver was extracted by the commercial kit RNAiso PLUS produced by TaKaRa. RNA was quantified using a Nanodrop 2000 UV-spectrophotometer. The genomic DNA was eliminated by the incubation of total RNA with the Eraser of gDNA. RNA was reverse transcribed with the PrimeScript™ RT reagent kit for real-time PCR. Specific primers for qPCR were designed by primer 6.0 (Table 2). SYBR® Green from Vazyme was used for the qPCR amplification on a quantitative thermal cycler (LightCycler 480 II, Switzerland). The qPCR program was as follows: 95°C for 3 min, then 40 cycles of 95°C for 12 s, 57°C for 12 s, and 72°C for 25 s. Comparative 2^−△△Ct^ method was used in the analysis of relative fold changes of genes which were normalized to the reference gene 18S rRNA.

#### 2.4.4. Analysis of Enzyme Activity

The liver tissue of common carp was homogenized and centrifuged according to the description of the kits. The collected supernatant was immediately used for enzyme activity analysis. Lipoprotein lipase (LPL), fatty acid synthase (FAS), and PEPCK activities were measured with commercial kits (Jiancheng Institute of Biological Engineering, Nanjing, China).

#### 2.4.5. Histopathological Detection of Liver

The fixed samples of the liver were dehydrated as per the standard protocol. The samples were then processed into the embedded wax blocks. It was cut into 4~5 mm sections. The sections were stained using hematoxylin and eosin (H&E) after drying. The liver cells were imaged in a microscope scopeA1 (Zeiss, Germany).

#### 2.4.6. Microbiota Diversity Analysis

The gut microbiota profiling was done by Majorbio Bio-Pharm Technology. The DNA was extracted by using the fresh faecal samples with the E.Z.N.A stool DNA kit. The Illumina MiSeq platform was used in this study. The V3/V4 region of the 16S rRNA gene was sequenced. The assembled sequences were then curated using Mothur v1.31.2. and clustered into Operational Taxonomic Units (OTUs) at ≥97%.

### 2.5. Statistical Analysis

In this study, all obtained data was conducted in SPSS statistics version 22.0 (IBM, Michigan Avenue, USA). Normality and homogeneity of variances were checked. One-way analysis of variance (ANOVA) was used to analyze the obtained data. Duncan's multiple tests were determined to investigate the difference. The differences indicated significance when *P* < 0.05. All results values are presented as the means ± S.E. (standard error of the mean). Spearman's correlation coefficient tests were used for statistical analysis.

## 3. Results

### 3.1. The Function of Dietary Bile Acids (BAs) on Fish Growth

After 56 days of the feeding experiment, the final body weight (FBW) and specific growth rate (SGR) were significantly reduced in the high-fat diet group fish (HF) (*P* < 0.05) in Table 3. However, the decrease in growth performance parameter was attenuated by supplementation of BAs in a high-fat diet (Table 3), with no significant difference to the control diet group. The HF diets enhanced the hepatosomatic index (HSI) and viscerosomatic index (VSI) in comparison with that of the control group and the BAs diets group (*P* < 0.05; Table 3). No significant difference was observed in survival rates (SR) among different treatments (*P* > 0.05; Table 3).

### 3.2. Effect of Dietary BAs on Whole Fish Body Composition

The results of the composition of the whole fish body and muscle were shown in Table 4. The crude fat content in the HF group was significantly higher than that in the control group in the whole fish (*P* < 0.05; Table 4); however, no significant difference with the other two groups was determined in the BA group (*P* > 0.05; Table 4). Compared with the high-fat group, the high-fat supplementation with bile acids significantly improved the moisture content of the whole fish (*P* < 0.05; Table 4) and significantly increased the dry matter content compared with the control group (*P* < 0.05; Table 4). No significant statistical difference was observed in the composition of muscle among the three treatments.

### 3.3. The Function of Dietary BAs on Biochemical Parameters in Serum and Liver

The changes of biochemical values in serum and liver were comparatively analyzed among the three groups, and the results were presented in Table 5. After the feeding trial, serum glucose in the BA group was significantly lower than that in HF, which was not different from CO (*P* < 0.05; Table 5), while the hepatic glycogen in the BA group was significantly higher than that in the CO group. The content of TG, LDL-C, and TBA in the liver was significantly enhanced in the HF group (*P* < 0.05; Table 5). However, the supplementation of 60 mg/kg bile acids to the HF diets resulted in a certain alleviating effect, in which TG was significantly less than that of the HF group (*P* < 0.05; Table 5). Similar results of the effect of BAs on lipid parameters were shown in hepatic indexes (Table 5). No significant difference was found in the TBA level in the serum (*P* > 0.05; Table 5), but the TBA in the liver of the HF group was higher than that both in the CO and BA group (*P* < 0.05; Table 5).

### 3.4. Effect of Dietary BAs on Hepatic Histological Observation

**Figure 1:**
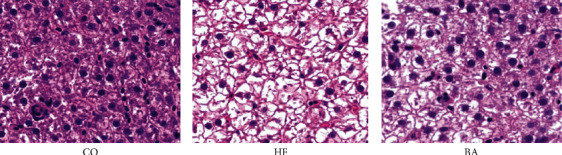
Histology of liver (H&E stain) of common carp fed with control (a) high-fat diet (b) and the supplementation with bile acid (c) diets for 8 weeks. In the HE staining of the histopathological sections of the three treatment groups, the typical morphology was observed and photographed under a 400x microscope. Bar: 20 *μ*m.

The histology of the liver was shown in Figure 1. Interestingly, there was a serious increase in fat vacuolization, the nuclear was migrated, and the number of nuclei in the liver was reduced in fish fed on HF diets. However, the combined administration of 60 mg/kg BAs significantly reduced the size of hepatic adipocytes compared with the high-fat group.

### 3.5. Effect of Dietary BAs on Enzyme Activity

The enzyme activities of LPL, FAS, and PEPCK in the HF group were higher than those in the CO group (*P* < 0.05; Table 6). The administrations of BAs significantly enhanced the activity of the LPL compared with the HF group. However, the activity of PEPCK in the BA group was lower than in the HF group (*P* < 0.05; Table 6).

### 3.6. The Function of Dietary BAs on the Gene Expression Involved in Lipid Metabolism, Carbohydrate Metabolism, and Cholesterol Metabolism

**Figure 2:**
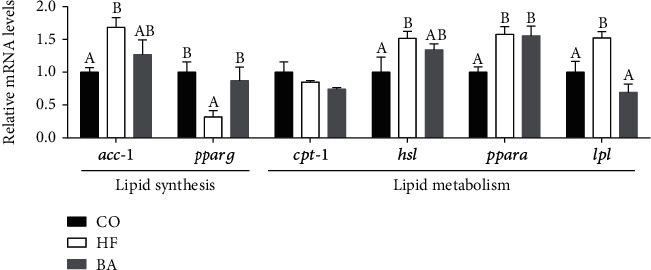
Relative mRNA expression levels of genes involved in lipid synthesis and metabolism in the dietary liver of common carp fed with test diets. Values are means ± S.E. per diet in triplicate tanks (*n* = 3). The different letters indicate differences between diets (one-way ANOVA analysis, Duncan's test, *P* < 0.05).

The transcript level of genes involved in lipid synthesis (ACC-1, PPARG) and lipid metabolism (CPT-1, HSL, PPAR*a*, and LPL) was presented in Figure 2. Long-term feeding with high lipid diet resulted in higher expression of ACC-1, HSL, PPAR*a*, and LPL than that in the CO group but lower expression of PPARG than that in the CO group. Dietary supplementation with BAs significantly increased the mRNA level of PPARA (*P* < 0.05; Figure 2). PPARG was also significantly upregulated in the BA group compared to the HF group, but LPL was downregulated after supplementation with BAs. There was no significant difference between the BA group and the control group in ACC-1, PPARG, HSL, and LPL (*P* > 0.05, Figure 2).

**Figure 3:**
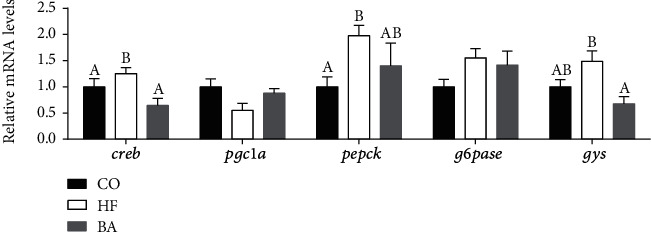
Relative mRNA expression levels of sugar and metabolism-related genes in livers of common carp fed with test diets. Values are means ± S.E. per diet in triplicate tanks (*n* = 3). The different letters indicate differences between diets (one-way ANOVA analysis, Duncan's test, *P* < 0.05).

Regarding carbohydrate metabolism-related genes, the expression of CREB, PEPECK, and GYS in common carp was found elevated in the HF group (*P* < 0.05; Figure 3), but after bile acid supplementation, the upregulation effect of these genes was alleviated. Among them, CREB and PEPCK were remarkably downregulated in the BA group by comparison to the high lipid group (*P* < 0.05; Figure 3).

**Figure 4:**
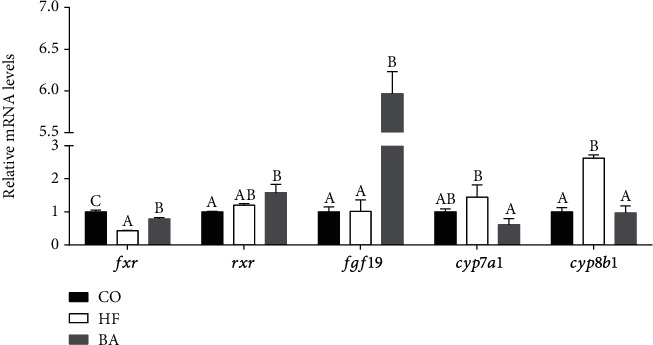
Relative mRNA expression levels of the cholesterol metabolism in the livers of common carp fed with test diets. Values are means ± S.E. per diet in triplicate tanks (*n* = 3). The different letters indicate differences between diets (one-way ANOVA analysis, Duncan's test, *P* < 0.05).

In the cholesterol metabolism-related genes, the expression of fxr was significantly downregulated, while cyp8a1 was upregulated (*P* < 0.05; Figure 4). Contrarily, after supplementation of BAs, the upregulation of FXR and FGF19 was observed in the high lipid diet, but downregulation of CYP7A1 and CYP8B1 was found compared to that in the high lipid group (*P* < 0.05; Figure 4). Moreover, RXR and FGF 19 in the BA group were remarkably higher than that in the CO group (*P* < 0.05; Figure 4).

### 3.7. Effect of Dietary BAs on Intestinal Microbiota

**Figure 5:**
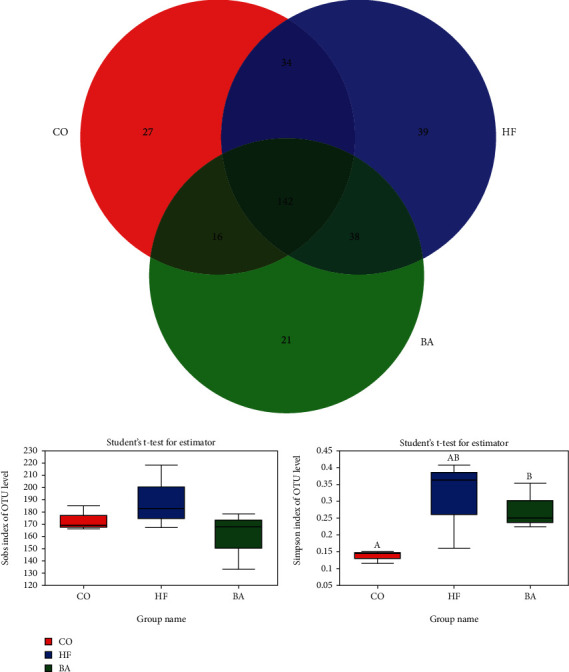
Wayne's diagram, Sobs' index, and Simpson's index at the OTU level in common carp fed with test diets. Wayne's plots represent the phylum-level number of gut microbiota in the three groups. The Sobs index of OTU levels was compared among three groups, by *t*-test. The Simpson index at the OTU levels was compared among the three groups, and the asterisks indicate significant differences by a *t*-test (*P* < 0.05).

**Figure 6:**
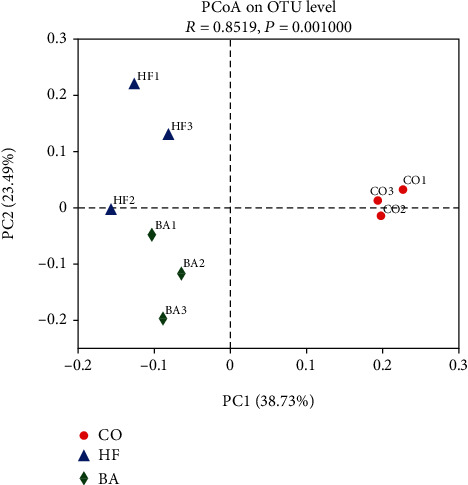
Principal coordinate analysis (PCoA) based on unweighted UniFrac distance matrices at the OTU level in common carp fed with test diets.

A total of 440076 effective sequences were obtained at the 97% similarity level from the samples. The average read length was 416 bp. In the three experimental treatments, a total of 142 species OTUs were shared between each group (Figure 5). The Venn diagram results also show that the unique OTUs in the high lipid group were the highest among all the three groups. Alpha diversity indices such as the Sobs index and the Simpson indexes were presented in Figure 5. It revealed that there were some differences in the Simpson index of the microbial community in the study group after supplementary with BAs (*P* < 0.05). Principal coordinate analysis (PCoA) based on unweighted UniFrac distance matrices demonstrated that the high lipid group and the BA-treated group were isolated from that of the control group (Figure 6).

**Figure 7:**
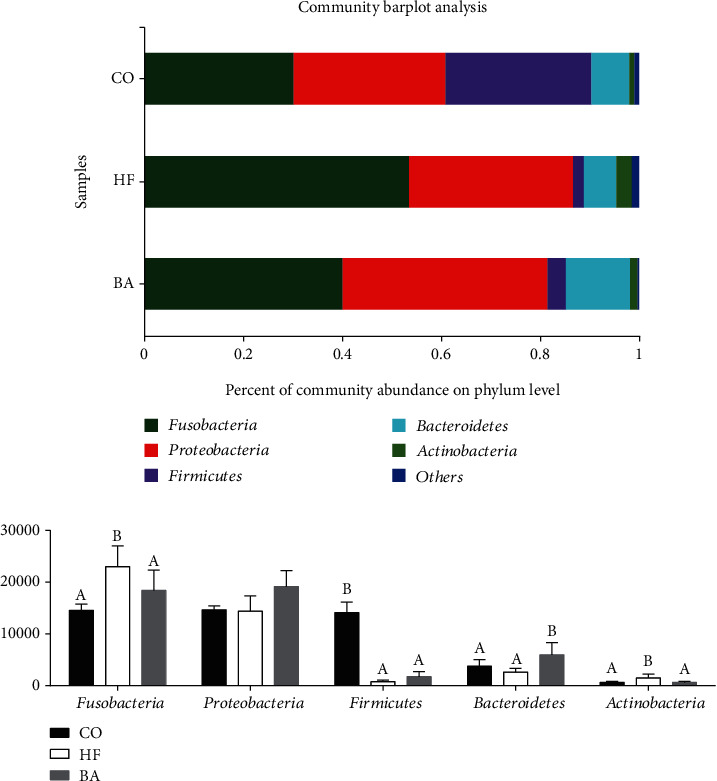
The intestinal microflora composition of the carp fed with test diets at the phylum level. Values are means ± S.E. per diet in triplicate tanks (*n* = 3). The different letters indicate differences between diets (one-way ANOVA analysis, Duncan's test, *P* < 0.05).

At the bacterial phyla level, five phyla, *Fusobacteria*, *Proteobacteri*a, *Firmicutes*, *Bacteroidetes,* and *Actinobacteria* were detected in intestinal bacterial communities of the all-experiment group (Figure 7). The results suggested that the dominant phylum in the common carp intestine is *Fusobacteria* and *Proteobacteria*, with more than 60% (Figure 7). The abundance of *Firmicutes* was markable decreased in the HF group and BA group (*P* < 0.05). There was significantly change in the abundance of *Fusobacteria* and *Actinobacteria* in the HF group, which were significantly elevated (*P* < 0.05). Moreover, the percentage of *Bacteroidetes* was significantly elevated in the BA group (*P* < 0.05).

### 3.8. Correlation Analysis

**Figure 8:**
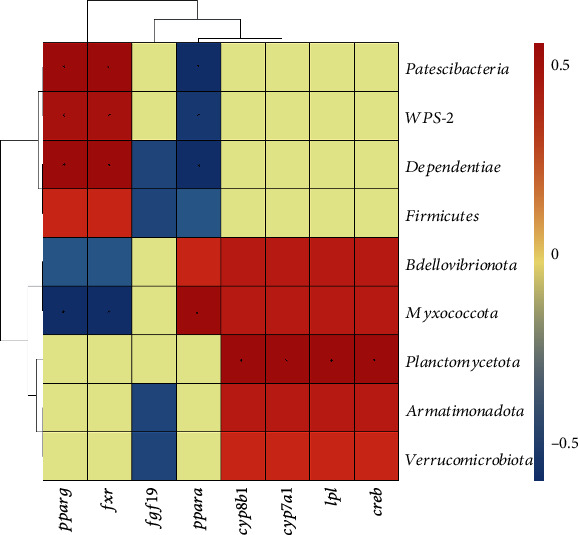
The Spearman correlation analysis between differential genes and the phylum level of gut microbiota in common carp. Resulted in a heatmap with red indicating positive, blue indicating negative, and white without *P* > 0.05, ^∗^*P* < 0.05.

The relationships among gene expression results and the difference in the community of gut microbiota at the phylum level were evaluated (Figure 8). The results presented that phylum *Patescibacteria*, *WPS−2*, and *Dependentiae* were significantly (positively) associated with PPARG and FXR, but negatively related to PPARA (*P* < 0.05). On the contrary, *Myxococcota* were negatively correlated with PPARG and FXR but positively correlated with PPARA (*P* < 0.05). Moreover, the abundance of phylum *Planctomycetota* with the expression of CYP8B1, CYP7A1, LPL, and CREB was related positively (*P* < 0.05).

## 4. Discussion

After 8 weeks of the feeding trial, this study found that growth indices such as FBW and SGR were decreased by feeding with high-fat diets. Similar results were observed in common carp (*Cyprinus carpio* L.) and meagre (*Argyrosomus regius*) [30, 31]. The high-fat content in the feed exceeding the physiological requirements of the animal may cause a digestive disturbance, reduction in feed intake, and downregulation of the absorption and utilization of nutrients, which ultimately results in growth performance impairment [32–34]. In the current study, we observed that the bile acid supplementation could effectively alleviate the negative effects of a high-fat diet in common carp. Zhou et al. [18] also found that the growth of grass carp in the high fat-added BA group was significantly higher. Similar results were reported in *Micropterus salmoides* and large yellow croaker (*Larimichthys crocea*) [17, 35] supplemented with BAs.

In the present study, the hepatosomatic index (HIS) was significantly increased in the high-fat group. Consistent results were also reported in tiger puffer, largemouth bass (*Micropterus salmoides*), and large yellow croaker [16, 17, 35]. Although the effect of BAs on the reduction of the HSI has been reported in tiger puffer and large yellow croaker [16, 35], the HSI was not significantly lower in the treatment of BAs in our study. Consistent results have also been reported for *Micropterus salmoides* [17]. The treatment effect of BAs seems different among species.

In the current study, the crude fat content of whole fish was significantly increased after dietary high-fat feed but with no influence on the crude protein content in the whole body and muscle. This is reflected in a positive correlation between crude fat level in the whole body and fat content consumed [36]. Similarly, the high body crude fat content induced by a high-fat diet was found in grass carp [34, 36] and tilapia [37]. Interestingly, there was no statistically significant change in crude fat content between the bile acid-supplemented group (BA) and the CO group. Our results were in agreement with that of *Micropterus salmoides* [17] and *Ctenopharyngodon idella* [18]. Therefore, the addition of bile acids to feed could improve the content of crude fat in fish.

Generally, excessive lipids in the body could increase fat deposition in the visceral cavity or mesentery, including the liver, adipose tissue, and the muscle [36, 38–40]. The current study indicated that a high-fat diet leads to fat vacuolization in the liver and increases in blood glucose and TG levels. Li et al. [34] also demonstrated that grass carp fed a high-fat diet (107.9 g kg^−1^ lipid) showed higher serum glucose and TG. After BAs supplementation, the hyperlipidemia was relieved, and the blood glucose and TG returned to the same significant difference as the CO group. The positive effect of BAs on serum lipids has also been described in largemouth bass and tiger puffer [16, 17]. Although LDL-C was not affected by BAs in largemouth bass and grass carp, our results indicated that LDL-C was significantly lower in the BA group, which is in agreement with that in tiger puffer [16]. The function of bile acids on blood and liver indices may be different among species.

To explore the mechanism of glucose and lipid metabolism, the transcript levels of genes involved in glucose and lipid metabolism in the liver were analyzed. The transcript level of PEPCK (a key enzyme of gluconeogenesis) was significantly elevated in the high-fat group, which might contribute to the high value of serum glucose. After supplementation with bile acids, the expression of PEPCK and CREB was downregulated, with no remarkable difference from the control group. Similar results were obtained for the enzyme activity of PEPCK. It seems that the expression of PEPCK and CREB was regulated by bile acids, which then downregulated the serum glucose.

The mRNA abundance of lipid metabolism (HSL, LPL) genes was enhanced in fish fed the high-fat diet in the present study. LPL is a modulator of tissue fatty-acid uptake in animals [41, 42]. FAS activity was also notably enhanced in the HF group. It is suggested that the increase in liver TG may arise from the uptake of fatty acids. Yuan et al. [36] also reported similar results in grass carp. Moreover, the transcript of PPAR*α* was significantly enhanced in the high-fat group and BA group. This is consistent with the results for grass carp [34] and Atlantic salmon (*Salmo salar* L.) [43]. Mitochondrial lipid oxidation may be enhanced by a high-fat diet. The high expression of PPA*rα* in the BA group was also reported in grass carp [18]. The effect of bile acids on fat degradation may be regulated by promoting PPAR*α* and fat decomposition.

Previous reports have indicated that cholesterol 7a-hydroxylase (CYP7A1) and CYP8B1 could elevate hepatic cholesterol synthesis rates and double the total bile acid pool size [44]. Consistent with these results, the higher expression of CYP7A1 and CYP8B1 in the high-fat group could increase the TBA in the liver almost 1.46-fold relative to the control group in the present study. Similarly, a high-fat diet can cause pathological changes in mouse liver cells, which will induce excessive expression of TBA in the liver [45]. However, supplementation with BAs resulted in suppression of the expression of CYP7A1 and CYP8B1 and a decrease of TBA in the liver. A similar result was reported in primary hepatocyte cultures [46]. However, there was no function of dietary BAs in the transcription of these genes in tiger puffer [16]. The function of bile acids on TBA may be caused by reducing the mRNA levels of the liver CYP7A1 and CYP8B1. However, this role may be species specific.

Interestingly, the transcript level of FGF19 was markedly higher in the BA group. FGF19 regulates glucose and lipid metabolism mainly through the inhibition of gluconeogenesis and the promotion of glycogen synthesis [22]. We also observed that serum glucose decreased and liver glycogen deposition increased in the bile acid addition group. Similar results were observed in intestinal cells [47]. The upregulation of FGF19 expression may affect its participation in inhibiting gluconeogenesis and promoting glycogen synthesis in fish.

High-fat diets not only affected the physiological and biochemical indices in serum and the liver but also changed the gut microbiota. In the present study, the OTU in the high-fat group was the highest. This result is similar to a report on grass carp [18, 48]. The OTU was lower after supplementation with bile acids. Bile acids can interact with phospholipids in the membrane of bacterial cells, which exert antimicrobial activity by damaging the bacterial membranes [49]. In addition, these increased OTUs in the high-fat group might most likely be derived from the phyla *Fusobacteria* or *Actinobacteria*. *Fusobacteria* is a gram-negative bacterium and the dominant bacterium in fish intestinal microbiota [48, 50] and is the agent of some fish bacterial diseases [51]. Although *Actinobacteria* occurs only as a small percentage of the microbiota, it plays an important role in the stability of the gut microbiome [52]. After supplementation with bile acids in a high-fat diet, *Bacteroidetes* were significantly enriched in this study. *Bacteroidetes* are gram-negative bacteria, which are considered beneficial to fish health and participate in sophisticated intestinal microbiota homeostasis. A similar result was reported in grass carp fed diets with bile acids [18].

Therefore, a high-fat diet resulted in a change in the intestinal microbiota, especially with bile acids, which could reshape the gut microbiota. The results obtained by the Spearman correlation analysis indicate that *Patescibacteria*, *Dependentiae*, *Myxococcota*, and *Planctomycetota* in the gut are associated with genes involved in glucose and lipid metabolism. Among them, *Patescibacteria* is distributed in adipose depots in humans, which might be associated with obesity [53]. It is indicated that there might be a relationship between gut flora and the gene expression changes in glycolipid metabolism. The association might be used as a biological indicator to indicate glycolipid metabolism disorder.

## 5. Conclusions

In conclusion, when common carp are under a high lipid diet load, growth is inhibited, and liver lipid accumulation is increased. The addition of bile acids (60 mg/kg) to the high-fat diet could promote the growth of common carp, alleviate hepatic lipid accumulation, and enhance the transcript level of lipolysis genes but reduce the mRNA level of gluconeogenesis genes and cholesterol synthesis genes. Moreover, bile acids remodel the gut flora, with *Bacteroidetes* significantly enriched, and some gut flora are correlated with the expression of glucose and lipid metabolic genes.

## Figures and Tables

**Table 1:** Formulation of the experimental diets (g/kg).

Ingredients	CO	HF	BA
Fish meal	16	16	16
Meat and bone meal	4	4	4
Corn gluten meal	28	28	28
Wheat grain	15	15	15
Corn starch	14.4	14.4	14.4
Cellulose microciystalline	15.6	9.9	9.84
Choline chloride	0.5	0.5	0.5
Carboxy methyl cellulose	1.5	1.5	1.5
Corn oil	1.095	6.795	6.795
Linseed oil	0.905	0.905	0.905
Vitamin premix^1^	1	1	1
Mineral premix^1^	2	2	2
Bile acids^2^	0	0	0.06
Proximate composition (% dry matter)			
Dry matter	93.9	93.3	93.4
Crude protein	32.5	32.5	32.5
Crude lipid	5.4	11.0	11.0
Ash	10.1	10.4	10.3

^1^Vitamin premix and mineral premix, designed for marine finfish, were purchased from Qingdao Master Biotech Co., Ltd., Qingdao, China. ^2^Hyodeoxycholic acid 67~70%, chenodeoxycholic acid 17~20%, hyocholic acid 8 ~ 10%, and trace ursodeoxycholic acid and binding bile acid.

**Table 2:** Primer sequences for the quantitative real-time PCR.

Gene	Forward and reverse primer (5′-3′)	GeneBank accession no.
LPL F	CGCTCCATTCACCTGTTCAT	FJ716101.1
LPL R	GCTGAGACACATGCCCTTATT	
HSL F	ATGATTTGGATGCGCAGACC	MF061228.2
HSL R	AAACGCTCCAGTGCAGTTTG	
CPT-1b F	CAGATGGAAAGTGTTGCTAATGAC	JQ361077.1
CPT-1b R	TGTGTAGAAGTTGCTGTTGACCA	
PPAR*α* F	GCGTGCTTTGGCTTTGTT	FJ849065.1
PPAR*α* R	GGGAAAGAGCAGCACGAG	
PPAR*γ* F	AAGTCACCGAATTCGCCAAG	FJ849064.1
PPAR*γ* R	TGCCGTCTTTGTTCATGAGG	
ACC-1 F	GTCACTGGCGTATGAGGATATT	XM_042757417.1
ACC-1 R	TCCACCTGTATGGTTCTTTGG	
CYP7A1 F	CTACCTGACCCTCTTTGG	XM_042718130.1
CYP7A1 R	CGTGGAGCATTGTCTTGG	
CYP8B1 F	CTTGCCATTGCTTGTCAT	XM_019073965.2
CYP8B1 R	GTATTCGGCTATCGTTCC	
RXRA F	CACCCAATGATCCAGTCACAAACA	XM_019094166.2
RXRA R	AGCTCATTCCATCCTGCTCGTAGA	
CREB F	CTCAGCAGATTGCCACCTTGG	XM_042716441.1
CREB R	GGGCAGCTGAACTAAGGTCAC	
PGC1A F	TGCCTGAGCTTGACCTCTCT	XM_042761149.1
PGC1A R	CGTCTTCATCCACTGGGATAC	
FGF19 F	GTGGGAAAACTGTATGGATCGC	XM_042728520.1
FGF19 R	AAGGGCTCCATGTTTGCTTG	
FXR F	CCCCTACAGCCATCAGT	XM_042729372.1
FXR R	GACCGACAATGCTCCCT	
G6Pase F	GAGGCCTTCAACAGACAGAAA	XM_042767066.1
G6Pase R	GAGCTTTGAGAAGCAGGTACAA	
GYS F	TTTTGGCCGCTGGTTGATTG	XM_042722011.1
GYS R	ATAGGGTAGTCCAATGCTGCAC	
PEPCK F	ACTCTTTGGGCAGACCTTTAC	XM_042751860.1
PEPCK R	CTGTCTGGTGTCAGGAAGATG	
18S F	GAGACTCCGGCTTGCTAAAT	FJ710826.1
18S R	CAGACCTGTTATTGCTCCATCT	

**Table 3:** Growth performance and biological parameters of common carp fed with test diets for 8 weeks.

	CO	HF	BA
IBW (g)	6.34 ± 0.02	6.34 ± 0.01	6.33 ± 0.01
FBW (g)	15.32 ± 0.11^b^	14.96 ± 0.07^a^	15.05 ± 0.06^ab^
WGR (%)	141.47 ± 2.31	135.82 ± 1.01	137.55 ± 1.16
SGR (%/day)	1.57 ± 0.02	1.53 ± 0.01	1.55 ± 0.01
CF (g/cm^3^)	2.42 ± 0.01^a^	2.99 ± 0.04^b^	2.78 ± 0.18^b^
HSI (%)	1.98 ± 0.04^a^	2.91 ± 0.04^c^	2.42 ± 0.05^b^
VSI (%)	6.62 ± 0.08^a^	8.12 ± 0.11^c^	7.23 ± 0.16^b^
SR (%)	96.67 ± 1.67	95.00 ± 2.89	98.33 ± 1.67

IBW and FBW: initial and final body weight; WGR: weight gain rate; SGR: specific growth rate; CF: condition factor; HSI: hepatosomatic index; VSI: viscerosomatic index; SR: survival rate. Mean values ± S.E. per diet in triplicate tanks (*n =9*). Different letters indicate differences between diets (one-way ANOVA analysis, Duncan's test, *P* < 0.05).

**Table 4:** Body composition of common carp fed with test diets for 8 weeks.

	CO	HF	BA
Whole fish (%)			
Moisture	73.90 ± 0.18^b^	72.89 ± 0.26^a^	74.35 ± 0.35^b^
Crude protein	13.54 ± 0.24	12.17 ± 0.90	11.51 ± 0.79
Crude fat	7.05 ± 0.72	8.96 ± 0.43	8.27 ± 0.15
Ash	3.18 ± 0.11	3.92 ± 0.36	3.65 ± 0.08
Muscle (%)			
Moisture	79.93 ± 0.10	79.83 ± 0.11	80.16 ± 0.33
Crude protein	15.81 ± 0.60	15.55 ± 0.39	15.38 ± 0.36
Crude fat	2.22 ± 0.06	2.49 ± 0.17	2.21 ± 0.04
Ash	1.22 ± 0.05^a^	1.27 ± 0.01^a^	1.52 ± 0.06^b^

Mean values ± S.E. per diet in triplicate tanks (*n* = 6). Different letters indicate differences between diets (one-way ANOVA analysis, Duncan's test, *P* < 0.05).

**Table 5:** Serum and liver biochemical indicators of common carp fed with test diets for 8 weeks.

Index	CO	HF	BA
Serum			
Glucose (GLU, mmol/L)	4.32 ± 0.41^a^	6.94 ± 0.33^b^	4.86 ± 0.71^a^
Triglycerides (TG, mmol/L)	2.89 ± 0.22^a^	4.03 ± 0.21^b^	3.38 ± 0.38^ab^
Total cholesterol (T–CHO, mmol/L)	2.96 ± 0.27^a^	3.85 ± 0.25^b^	3.31 ± 0.22^ab^
High density lipoprotein cholesterol (HDL–C, mmol/L)	1.83 ± 0.06	1.92 ± 0.10	1.86 ± 0.03
Low density lipoprotein cholesterol (LDL–C, mmol/L)	0.69 ± 0.03^b^	0.66 ± 0.01^ab^	0.62 ± 0.022^a^
Total bile acid (TBA, *μ*mol/L)	7.45 ± 0.24	7.06 ± 0.37	8.00 ± 0.27
Liver			
Triglycerides (TG, mmol/g)	13.96 ± 0.94^a^	18.85 ± 0.48^b^	15.43 ± 0.91^a^
Total cholesterol (T-CHO, mmol/g)	4.20 ± 0.31	4.63 ± 0.18	4.08 ± 0.22
High density lipoprotein cholesterol (HDL-C, mmol/g)	2.46 ± 0.31	3.02 ± 0.23	2.46 ± 0.27
Low density lipoprotein cholesterol(LDL-C, mmol/g)	1.48 ± 0.03^a^	2.96 ± 0.22^b^	2.17 ± 0.33^ab^
Total bile acid(TBA, *μ*mol/L)	13.75 ± 0.41^a^	20.16 ± 0.66^b^	15.24 ± 0.81^a^
Glycogen (LG mg/g)	0.29 ± 0.01^a^	0.31 ± 0.01^ab^	0.33 ± 0.01^b^

Values are means ± S.E. per diet in triplicate tanks (*n* = 6). The different letters indicate differences between diets (one-way ANOVA analysis, Duncan's test, *P* < 0.05).

**Table 6:** Key enzymatic activities of glucose and lipid metabolism in liver of common carp fed with test diets.

	CO	HF	BA
Lipoprotein lipase (LPL, U/g)	33.99 ± 0.34^a^	39.97 ± 0.25^b^	48.66 ± 0.20^c^
Fatty acid synthase (FAS, nmol/min/g)	294.44 ± 1.13^a^	366.43 ± 2.36^b^	363.33 ± 5.26^b^
Phosphoenolpyruvate carboxykinase (PEPCK, U/g)	571.52 ± 32.39^a^	798.55 ± 32.04^b^	642.06 ± 31.63^a^

Mean values ± S.E. per diet in triplicate tanks (*n* = 9). Different letters indicate differences between diets (one-way ANOVA analysis, Duncan's test, *P* < 0.05).

## Data Availability

On a reasonable suggestion, the corresponding author will provide the information data supporting the study's conclusions.
